# Characterization of Bacteriophage Peptides of Pathogenic *Streptococcus* by LC-ESI-MS/MS: Bacteriophage Phylogenomics and Their Relationship to Their Host

**DOI:** 10.3389/fmicb.2020.01241

**Published:** 2020-06-09

**Authors:** Ana G. Abril, Mónica Carrera, Karola Böhme, Jorge Barros-Velázquez, Benito Cañas, Jose L. R. Rama, Tomás G. Villa, Pilar Calo-Mata

**Affiliations:** ^1^Department of Microbiology and Parasitology, Faculty of Pharmacy, University of Santiago de Compostela, Santiago de Compostela, Spain; ^2^Department of Food Technology, Spanish National Research Council, Marine Research Institute, Vigo, Spain; ^3^Agroalimentary Technological Center of Lugo, Lugo, Spain; ^4^Department of Analytical Chemistry, Nutrition and Food Science, School of Veterinary Sciences, University of Santiago de Compostela, Lugo, Spain; ^5^Department of Analytical Chemistry, Complutense University of Madrid, Madrid, Spain

**Keywords:** pathogen detection, LC-ESI-MS/MS, proteomics, mass spectrometry, phage peptide biomarker

## Abstract

The present work focuses on LC-ESI-MS/MS (liquid chromatography-electrospray ionization-tandem mass spectrometry) analysis of phage-origin tryptic digestion peptides from mastitis-causing *Streptococcus* spp. isolated from milk. A total of 2,546 non-redundant peptides belonging to 1,890 proteins were identified and analyzed. Among them, 65 phage-origin peptides were determined as specific *Streptococcus* spp. peptides. These peptides belong to proteins such as phage repressors, phage endopeptidases, structural phage proteins, and uncharacterized phage proteins. Studies involving bacteriophage phylogeny and the relationship between phages encoding the peptides determined and the bacteria they infect were also performed. The results show how specific peptides are present in closely related phages, and a link exists between bacteriophage phylogeny and the *Streptococcus* spp. they infect. Moreover, the phage peptide M^∗^ATNLGQAYVQIM^∗^PSAK is unique and specific for *Streptococcus agalactiae*. These results revealed that diagnostic peptides, among others, could be useful for the identification and characterization of mastitis-causing *Streptococcus* spp., particularly peptides that belong to specific functional proteins, such as phage-origin proteins, because of their specificity to bacterial hosts.

## Introduction

*Streptococcus* spp. are among the main mastitis pathogens present in dairy products (Forsman et al., 1997; Böhme et al., 2012). The genus *Streptococcus* includes numerous mastitis-causing species that are responsible for high economic losses as well as human health issues (Lopez-Sanchez et al., 2012; Richards et al., 2014). The major species involved in both clinical and subclinical mastitis are *Streptococcus agalactiae*, *Streptococcus canis*, *Streptococcus dysgalactiae* and *Streptococcus uberis* (Lundberg et al., 2014; Richards et al., 2014). Additionally, *Streptococcus gallolyticus* (Dumke et al., 2015) and *Streptococcus parauberis* (Park et al., 2013) have been reported as minor mastitis agents.

It is well known that *Streptococcus* spp. may carry temperate bacteriophages in their genomes (Brüssow and Desiere, 2001; Romero et al., 2004; Fortier and Sekulovic, 2013). These phages are usually integrated into bacterial chromosomes as prophages, wherein they may provide new and beneficial properties to the host, or in contrast, they may disrupt genes, thus affecting their expression (Fortier and Sekulovic, 2013). Phage genome excisions and integrations are mediated by phage-encoded DNA recombinases (Menouni et al., 2015), which can act at specific phage attachment sites in the bacterial genome that are identical to those present in the phage genome. Some phages can integrate randomly within the bacterial genome; for example, phage Mu (as long as a particular gene is not expressed). It is evident that the interaction of streptococcal species with bacteriophages may greatly alter the variability in bacterial populations (Feiner et al., 2015).

Only 3% of phage genomes in the NCBI nucleotide database represent active phages against *Streptococcus* spp. (Harhala et al., 2018). Some phages have been reported and described in *S. uberis* (Hill and Brandy, 1989), *S. agalactiae* (Domelier et al., 2009; Bai et al., 2013), and *S. dysgalactiae* (Davies et al., 2007) by means of different methods, such as molecular characterization and complete genome sequencing. There are some well-known *Siphoviridae* phages of *Streptococcus* spp., including the species Sfi21, Sfi11, and Sfi19, which are mainly found in *Streptococcus thermophilus* (Brüssow and Desiere, 2001; Canchaya et al., 2004). Additionally, the genome sequence of EJ-1, a *Myoviridae* phage of *Streptococcus pneumoniae*, has been reported (Romero et al., 2004).

The interaction between bacteria and phages, including pathogenic and industrially applicable bacteria, is a rapid coadaptation that allows highly dynamic coevolution (Koskella and Brockhurst, 2014). It is known that closely related phages normally occupy the same genome insertion site in different bacteria. A specific site can be occupied by a phage, empty in another strain or replaced by an altogether different phage (Canchaya et al., 2004).

Classic culture-based methods used for the detection of pathogenic bacteria (Ivnitski et al., 1999; Chakravorty et al., 2007) and their phages (Gantzer et al., 1998; Ackermann, 2007) are time-consuming and laborious procedures. Therefore, new rapid molecular microbial diagnostic methods based on genomics and proteomics tools have been developed to achieve faster and more efficient detection, identification and strain biotyping of bacterial species (Lasch et al., 2009; Böhme et al., 2011, 2012; Branquinho et al., 2014; Quintela-Baluja et al., 2014). As bacteriophages are highly specific for their host pathogens, phage typing is a classic technique used for bacterial detection and characterization (Craigie and Yen, 1938). Phage receptor-binding proteins (RBPs), as well as the study of their nucleic acids, the use of antibodies, and phage-display peptides (PDPs), have been studied as biosensors for pathogen detection (Rees and Voorhees, 2005; Lavigne et al., 2009; Singh et al., 2013; Chanishvili, 2015; Richter et al., 2018).

Proteomics techniques using matrix-assisted laser desorption/ionization time of flight mass spectrometry (MALDI-TOF MS) and liquid chromatography-electrospray ionization-tandem mass spectrometry (LC-ESI-MS/MS) instruments have been used for the analysis and detection of pathogenic bacterial strains based on specific diagnostic peptides (Calo-Mata et al., 2016; Pfrunder et al., 2016). In addition, LC-ESI-MS/MS techniques have been employed for the identification and detection of bacterial bacteriophages such as the bacteriophage lambda (Serafim et al., 2017). However, no study has been published for *Streptococcus* spp. phage detection and identification by LC-ESI-MS/MS so far without previously phage purification for the analysis. With this method, putative temperate phages in addition to virulent phages present in the analyzed strains were identified.

In this work, we aimed to study for the first time the proteomics of specific peptides of streptococcal species for the identification of both phage and bacterial strains by LC-ESI-MS/MS.

## Materials and Methods

In this study, tryptic digestion peptides from the mastitis-causing strain *Streptococcus* spp. isolated from milk were analyzed by LC-ESI-MS/MS. A total of 100 μg of protein extraction was digested with trypsin, cleaned on a C18 microSpin^TM^, and analyzed by LC-MS/MS. Proteomic data were processed by SEQUEST (Proteome Discoverer 1.4 package, Thermo Fisher Scientific) against bacteria in the UniProt/TrEMBL database. The overall time of culture and protein extraction and purification was 3 days (depending on the strain, the time of growth may have been 1 or 2 days, another day for protein extraction and an additional day for protein purification). Mass spectrometry analysis and peptide detection took 2–3 h, according to the methodology employed in the present work.

### Bacteria

A total of 14 *Streptococcus* spp. strains (Table 1) were used in this study. Five reference strains were obtained from different culture collections (ST1, ST2, ST3, and ST14 from the Spanish culture collection and ST4 and ST5 from the German culture collection). Additionally, 9 strains were isolated from the milk of mastitis cows at the LHICA (Laboratorio de Higiene, Inspección y Control de Alimentos) (ST6, ST7, ST8, ST9, ST10, and ST11) laboratory, Faculty of Veterinary Sciences, University of Santiago de Compostela, Spain. These strains were genetically identified as *Streptococcus* spp. based on the VITEK 2 system and 16S rRNA gene sequencing (Alnakip et al., 2014; Quintela-Baluja et al., 2014). The strains were reactivated in brain heart infusion (BHI, Oxoid Ltd., Hampshire, United Kingdom) and incubated at 31°C for 24 h. Bacterial cultures were then grown on plate count agar (PCA, Oxoid) at 31°C for 24 h.

**TABLE 1:** *Streptococcus* spp. strains used in this study; total peptides were identified by LC-ESI-MS/MS and number of phage peptides was analyzed by BLASTp.

Sample	Species	Strain	Source	NCBI accession number	Total peptides	Phage peptides
ST1	*Streptococcus uberis*	ATCC 19436	CECT 994	JN630842.1	254	13
ST2	*Streptococcus agalactiae*	CECT 183	Milk	KC510212.1	377	7
ST3	*Streptococcus dysgalactiae* subsp. *equisimilis*	CECT 926	ATCC 9542		242	7
ST4	*Streptococcus parauberis*	DSM 6631	Mastitis sample milk	NR_043001.1	204	6
ST5	*Streptococcus parauberis*	DSM 6632	Raw milk	JN630844.1	121	3
ST6	*Streptococcus agalactiae*	USC1-LHICA	Mastitis sample milk	KP001323.1	117	1
ST7	*Streptococcus agalactiae*	USC3-LHICA	Mastitis sample milk	KC510215.1	160	5
ST8	*Streptococcus uberis*	USC5-LHICA	Mastitis sample milk	KC510216.1	122	6
ST9	*Streptococcus dysgalactiae* subsp. *dysgalactiae*	USC13-LHICA	Mastitis sample milk	KC510218.1	221	2
ST10	*Streptococcus canis*	USC52-LHICA	Mastitis sample milk	KC510222.1	217	5
ST11	*Streptococcus uberis*	USC69-LHICA	Mastitis sample milk	KC510224.1	36	0
ST12	*Streptococcus gallolyticus* subsp. *gallolyticus*	USC83-LHICA	Mastitis sample milk	KC510227.1	143	4
ST13	*Streptococcus gallolyticus* subsp. *gallolyticus*	USC84	Mastitis sample milk	KC51022.8	136	6
ST14	*Streptococcus dysgalactiae* subsp. *dysgalactiae*	CECT758	Mastitis sample milk	KC51021.3	214	6

*ATCC, American Type Culture Collection; CECT, Spanish Type Culture Collection; DSM, German Type Culture Collection.*

### Protein Extraction

Protein extraction was carried out as described previously (Böhme et al., 2010a, b; Carrera et al., 2017). Briefly, a loop full of each bacterial culture was harvested, and the biomass was mixed with 100 μL of a solution containing 50% acetonitrile (50% ACN) (Merck, Darmstadt, Germany) and 1% aqueous trifluoroacetic acid (1% TFA) (Acros Organics, NJ, United States). After vortexing and centrifugation, the supernatant was treated with a solution of lysis buffer [60 mM Tris-HCl pH 7.5, 1% lauryl maltoside, 5 mM phenylmethanesulfonyl fluoride (5 mM PMSF) and 1% dithiothreitol (1% DTT)]. The supernatants were transferred to a new tube and then quantified using the bicinchoninic acid method (Sigma Chemical Co., United States). All analyses were performed in triplicate.

### Peptide Sample Preparation

Protein extracts were subjected to in-solution tryptic digestion (Carrera et al., 2013). A total of 100 μg of protein extract was dried under vacuum in a SpeedVac (CentriVap, Labconco Co., United States) and resuspended for denaturation in 25 μL of 8 M urea in 25 mM ammonium bicarbonate (pH 8.0). After 5 min of sonication, DTT was added to a final concentration of 10 mM, and the solution was incubated at 37°C for 1 h. Alkylation was performed with the addition of iodoacetamide to a final concentration of 50 mM, and the solution was incubated for 1 h at room temperature in darkness. Then, the sample was diluted fourfold with 25 mM ammonium bicarbonate, pH 8.0, to decrease the urea concentration. Finally, proteins were digested with trypsin (1:100 protease-to-protein ratio) (Promega, WI, United States) at 37°C overnight.

### Shotgun LC-ESI-MS/MS Analysis

Peptide digests were acidified with formic acid (FA), cleaned on a C18 MicroSpin^TM^ column (The Nest Group, Southborough, MA, United States) and analyzed by LC-ESI-MS/MS using a Proxeon EASY-nLC II Nanoflow system (Thermo Fisher Scientific, San Jose, CA, United States) coupled to an LTQ-Orbitrap XL mass spectrometer (Thermo Fisher Scientific) (Calo-Mata et al., 2016; Carrera et al., 2017). The peptide separation (2 μg) was performed on a reverse-phase (RP) column (EASY-Spray column, 50 cm × 75 μm ID, PepMap C18, 2 μm particles, 100 Å pore size, Thermo Fisher Scientific) with a 10 mm precolumn (Accucore XL C18, Thermo Fisher Scientific) using a linear 120 min gradient from 5 to 35% solvent B (solvent A, 98% water, 2% ACN, 0.1% FA; solvent B, 98% ACN, 2% water, 0.1% FA) at a flow rate of 300 nL/min. For ionization, a spray voltage of 1.95 kV and a capillary temperature of 230°C were used. Peptides were analyzed in positive mode from 400 to 1,600 amu (1 μscan), followed by 10 data-dependent collision-induced dissociation (CID) MS/MS scans (1 μscan) using an isolation width of 3 amu and a normalized collision energy of 35%. Fragmented masses were set in dynamic exclusion for 30 s after the second fragmentation event, and unassigned charged ions were excluded from MS/MS analysis.

### LC-ESI-MS/MS Mass Spectrometry Data Processing

The results from the LC-ESI-MS/MS spectra were searched against the *Streptococcus* UniProt/TrEMBL database (13,528 reviewed and 1,290,635 unreviewed protein sequence entries) using SEQUEST-HT (Proteome Discoverer 2.1, Thermo Fisher Scientific). The following constraints were used for the searches: semitryptic cleavage with up to two missed cleavage sites and tolerance windows of 1.2 Da for precursor ions and 0.6 Da for MS/MS fragment ions. The variable modifications that were allowed were as follows: (M^∗^) methionine oxidation (+15.99 Da), (C^∗^) carbamidomethylation of Cys (+57.02 Da) and acetylation of the N-terminus of the protein (+42.0106 Da). The database search results were subjected to statistical analysis with the Percolator algorithm (Käll et al., 2007). To validate the peptide assignments, the false discovery rate (FDR) was kept below 1%.

### Selection of Potential Peptide Biomarkers

To study specificity, we used the BLASTp algorithm on each peptide that was identified by LC-ESI-MS/MS to determine homologies and exclusiveness with protein sequences registered in the NCBI database (Altschul et al., 1990). To search for BLASTp parameters, we included and excluded *Streptococcaceae* taxa with the aim of identifying which peptides belong to *Streptococcus* spp. bacteriophages.

### Phage Genome Comparison and Relatedness

Genomes of all studied *Streptococcus* spp. phages were downloaded from the GenBank database and analyzed and compared with the server VICTOR (Virus Classification and Tree Building Online Resource^1^); the intergenomic distances were calculated, and a phylogenomic tree was constructed (Meier-Kolthoff and Göker, 2017).

Holin gene sequences of the studied bacteriophages from the GenBank database were compared. Sequences were aligned using Clustal_X software (Thompson et al., 1997), and distances were calculated according to Kimura’s two-parameter model (Kimura, 1980). The phylogenetic trees were inferred using maximum likelihood (ML) models (Saitou and Nei, 1987; Rogers and Wofford, 1998). MEGA 7.09 software (Kumar et al., 2016) was used for the analyses.

## Results

### *Streptococcus* spp. Proteome Repository

Protein mixtures from each of the 14 different *Streptococcus* spp. strains were digested with trypsin and analyzed by LC-ESI-MS/MS.

A total of 2,546 non-redundant peptides corresponding to 1,890 different annotated proteins were identified for all *Streptococcus* spp. strains. Among them, 69 different peptides were identified as phage peptides. These phage peptides were selected, and the BLASTp algorithm was used to determine the homology and exclusiveness of these peptides with protein sequences registered in the NCBI database (Altschul et al., 1990). For the BLASTp parameters, the term *Streptococcaceae* was included and excluded with the aim of finding peptides belonging to *Streptococcus* spp. bacteriophages. Supplementary Table S1 in Supplementary Data 1 summarizes the list of 65 possible specific streptococcal bacteriophage peptides, bacterial peptides with putative phage origin, and bacteria and phages with 100% homology with respect to the protein NCBI database. All streptococcal phage peptides with 100% homology were found to belong mainly to *Siphoviridae* (*Sfi21* and *Sfi11* genera) and to *Podoviridae* (unclassified genera). These *Siphoviridae* and *Podoviridae* family genomes are usually organized into functional modules such as lysogeny, DNA replication, packaging, morphogenesis and lysis (Lucchini et al., 1999b; Xia and Wolz, 2014).

**FIGURE 1:**
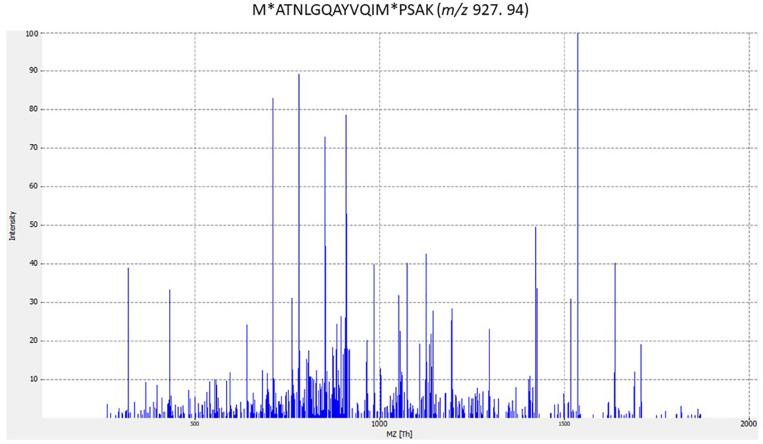
MS/MS spectrum for M^∗^ATNLGQAYVQIM^∗^PSAK species-specific peptide biomarker from *Streptococcus agalactiae*. The corresponding peptides were tested for specificity to S. *agalactiae* using the BLASTp algorithm. (M^∗^ methionine oxidation).

In some cases, peptides assigned to phage proteins did not correspond to any described phages in the NCBI database; for example, peptide LIDAELTKAGVRDAEIFGK from strain ST1 (Supplementary Table S1 in Supplementary Data 1). Furthermore, four phage peptides were identified only in streptococcal strains analyzed in this study but were not described in bacteria in the literature; for example peptide VKEKIAAIVNDEELLAK from strain ST1. Strikingly, 64 peptides found in this study were described for the first time in *Streptococcus* spp. analyzed; for peptide ITTGIISAARIGAEAITADKLK from strain ST1 (Supplementary Table S1 in Supplementary Data 1). This means that only one identified peptide was previously described from the species analyzed. Furthermore, the peptide M^∗^ATNLGQAYVQIM^∗^PSAK is highly specific to *S. agalactiae* in the NCBI database and *S. agalactiae* ST2 analyzed in this study (Supplementary Table S1 in Supplementary Data 1). Figure 1 shows the MS/MS spectrum for the M^∗^ATNLGQAYVQIM^∗^PSAK species-specific peptide biomarker from *S. agalactiae*.

### Phage Peptides Identified From the Analyzed *S. uberis* Strains

ST1, ST8, and ST11 were the strains analyzed for the species *S. uberis*. For strains ST1 and ST8, thirteen and five peptides were identified, respectively. The *S. uberis* tryptic peptides from strains ST1 and ST8 were found to possibly belong to twelve and twenty-five different described phages, respectively, based on a similarity search. The different peptides from strains ST1 and ST8 could belong to the same bacteriophage, P4761. The peptides identified in each strain and the bacteriophages in which the peptides were present based on similarity are shown in Supplementary Table S1 in Supplementary Data 1.

The majority of the determined ST1 peptides belonged to structural proteins such as head and tail proteins, a phage minor structural protein from the GP20 family, a Gp58-like protein and a tape measure protein. Gp58 is a minor structural protein previously described in *Lactobacillus delbrueckii* subsp. *lactis* bacteriophage LL-H (Mikkonen and Alatossava, 1994). The tape measure protein (TMP) determines the tail length and facilitates DNA entry into the cell during infection, and it has been reported that some TMPs carry lysozyme-like and peptidase domains (Rodriguez-Rubio et al., 2012; Mahony et al., 2016). A peptide from the RecT protein was also identified from an ST1 strain. RecT is a RecA-dependent, RecA-independent DNA single-strand annealing protein (SSAP) that belongs to the RecT/Redβ superfamily and is involved in DNA recombination pathways. All the mentioned SSAPs seem to be of bacteriophage origin; RecT proteins, on the other hand, have been described in several bacteria, while Redβ has only been found in lambdoid phages (Iyer et al., 2002; Menouni et al., 2015). Additionally, the phage endopeptidase and matrix-binding protein EbhB was determined from *S. uberis* strain ST1. It was reported that EbhB is an adhesin consisting of several domains encoding the largest protein in *Staphylococcus aureus* and is involved in agglutination, attachment to host extracellular matrix, biofilm accumulation, and escape from phagocytosis (Walker et al., 2013). For strain *S. uberis* ST8, the determined peptides belonged to a Gp10 family protein, a Gp348 protein, a phage integrase and two uncharacterized phage proteins. One of these uncharacterized phage peptides (Supplementary Table S1 in Supplementary Data 1) presents the same sequence as peptides from twenty-three different phages described in the NCBI database. As an identified protein in strain ST8, Gp10 is a putative tail component found in *Caudovirales*, and Gp348 is definitively the main capsid protein E (GpE); upon assembly, GpE acquires icosahedral symmetry, thus stabilizing the DNA in the head (Dokland and Murialdo, 1993).

### Determined Phage Peptides From the Analyzed *S. parauberis* Strains

ST4 and ST5 were the strains analyzed for the species *S. parauberis*. For strain ST4, four peptides were determined, and three peptides were determined for the ST5 strain. The peptides from *S. parauberis* strains ST4 and ST5 were found to belong to sixteen and five different described phages, respectively, as determined with the NCBI database. In summary, the determined peptides based on similarity for each strain and the bacteriophages in which these peptides are present are shown in Supplementary Table S1 in Supplementary Data 1.

Among the ST4-determined peptides, a phage peptide from a tail protein, an uncharacterized phage peptide and a peptide from a DUF1372 domain of unknown function were identified. It should be noted that there was a large number of uncharacterized protein sequences in databases and that more than 20% of all protein domains are annotated as “domains of unknown function” (DUFs) (Bateman et al., 2010; Goodacre et al., 2014). Several uncharacterized phage proteins and DUFs from *Streptococcus* spp. bacteriophages were identified from the analyzed strains (Bateman et al., 2010; Goodacre et al., 2014). The DUF1372 domain-containing proteins included several sequences belonging to *Streptococcus* spp. bacteriophage as well as other related proteins in *Streptococcus* spp. In addition, for strain ST4, a peptide from the GDSL-like family of lipases/acylhydrolases identical to some *Streptococcus* spp. phages was identified; GDSL-like family proteins are hydrolases that exhibit a variety of properties, including known broad substrate specificity and regiospecificity (Akoh et al., 2004).

For *S. parauberis* strain ST5, the determined peptides included a phage tail fiber PblB peptide (surface protein). In this sense, the surface proteins PblB and PblA, which encode streptococcal phages, are incorporated into phage particles and are involved in tail assembly, suggesting that both proteins are of bacterial prophage origin. These proteins were reported in *Streptococcus mitis* and increase platelet-binding ability (Bensing et al., 2001; Mitchell et al., 2007). Furthermore, PblA was also found in *Streptococcus oralis*, *Streptococcus pyogenes* and *S. pneumoniae.* However, both proteins, PblB and PblA, are uncommon since no known protein has strong similarity to these bacterial adhesins. For strain ST5, an uncharacterized protein peptide and peptides of the Cro/CI family of phage repressors were also identified. As integrases and regulator proteins, CI and Cro are encoded in the lysogeny module. If the CI repressor predominates, the phage will remain in the lysogenic state, but if Cro outnumbers CI, the phage will enter the lytic cycle. The most extended regulator in the prokaryotic world is the xenobiotic XRE, which contains a helix-turn-helix (HTH) conformation, is involved in DNA binding and shows similarity to the Cro repressor of phage λ (Durante-Rodríguez et al., 2016). In the NCBI database, peptides of the CI/Cro repressor types are usually named XRE-family proteins for bacteria.

### Phage Peptides Identified From the Analyzed *S. dysgalactiae* Strains

*Streptococcus dysgalactiae* strains ST3, ST9 and ST14 were analyzed in this study. For strains ST3 and ST9, seven and two peptides were identified, respectively. For ST14, five peptides were determined, and one of them could tentatively belong to two previously described phages. The different peptides from *S. dysgalactiae* strains ST3 and ST9 were found to belong to six and one different described phage, respectively, according to the NCBI database. In summary, the determined peptides from each strain and the bacteriophages in which the peptides are present, based on similarity, are shown in Supplementary Table S1 in Supplementary Data 1.

Four peptides identified from the ST3 strain belong to proteins of the structural module, including a major tail protein, a phage capsid protein, a head-tail connector and a phage assembly chaperone protein. This last protein is required in the morphogenesis of long-tailed phages (Pell et al., 2013). A phage integrase and a phage repressor of the Cro/CI type from the lysogeny module were also identified from the ST3 strain. Furthermore, a phage-like HicB family antitoxin was identified from this strain. This protein comes from the toxin–antitoxin loci that encode toxin and antitoxin proteins (the antitoxin has a DNA-binding domain and autoregulates the operon). This operon exhibits a complex phylogeny due to HGT (Horizontal gene transfer), and proteins of this family are often detected in phages, plasmids and bacterial genomes (Jørgensen et al., 2009).

The peptides of strain ST9 were identified as a prophage pi2 protein peptide from the morphogenesis module and an uncharacterized protein peptide. For ST14, a phage integrase family peptide, a phage tail tape measure peptide, a repressor of the Cro/CI type peptide and one uncharacterized protein peptide were determined by LC-ESI-MS/MS (Supplementary Table S1 in Supplementary Data 1).

### Phage Peptides Identified From the Analyzed *S. agalactiae* Strains

The strains analyzed for *S. agalactiae* were ST2, ST6, and ST7. For strain ST2, seven peptides were identified, and for ST6 and ST7, one and five peptides were determined, respectively (Supplementary Data 1). Additionally, *S. agalactiae* tryptic peptides from strains ST2 and ST7 were found to belong to seven and one different described phage, respectively. For *S*. *agalactiae* ST6, a phage peptide was determined, but it was not associated with a described characterized phage in the NCBI database.

Some of the identified peptides from ST2 belong to proteins of the morphogenesis module, including a phage tail protein, phage envelope protein and a minor phage structural protein. The identified peptide from the pblA tail fiber protein from strain ST2 is very specific for the species *S. agalactiae* (M^∗^ATNLGQAYVQIM^∗^PSAK) (Figure 1), in contrast to the analyzed peptide sequences that are present in streptococcal species (Supplementary Data 1). A phage replication protein peptide, phage-related chromosomal island protein (PrCI) peptide, and an uncharacterized protein peptide were also identified for strain ST2. PrCIs are characterized by a specific set of phage-related functions that allow transductional recombination events and carry genes involved in biofilm formation, superantigen synthesis, antibiotic resistance and phage resistance and encoding ATP-binding cassette (ABC) transporters. Several PrCIs have been identified in *S. aureus*, *S. pyogenes*, *Enterococcus. faecalis* and *Lactococcus lactis* (Novick et al., 2012). In addition, phages can carry CRISPR (clustered regularly interspaced short palindromic repeats) arrays that, with associated Cas proteins, target a chromosomal island of the bacterial host. *S. agalactiae* contains two CRISPR/Cas systems in its genome. One of them is present in several strains, and the other is ubiquitous. The *S. agalactiae* 2-A CRISPR1/Cas system preferentially targets ICE (integrative conjugative elements) and bacteriophages normally widespread among *S. agalactiae*. Likewise, *S. pyogenes* and *S. thermophilus* spacers match described sequences in viruses (Lopez-Sanchez et al., 2012; Koskella and Brockhurst, 2014).

For strain ST6, a peptide from an uncharacterized protein was identified. Identified peptides from strain ST7 belong to a major capsid protein, a tape measure protein and a phage integrase. Additionally, a DnaD and phage-associated domain-containing protein peptide and a tail fiber pblB peptide were identified in *S. agalactiae* ST7. This tail fiber pblB peptide was also identified in *S. parauberis* ST5, and this was the first time that this protein was identified in *S. agalactiae* and *S. parauberis* species. The aforementioned DnaD and phage-associated domain-containing proteins are DNA replication proteins that are involved in the primosomal step of DNA replication and DNA remodeling; these proteins can also bind to DNA and form large nucleoprotein complexes. DnaD-like motifs have been found in proteins of unknown function both in bacterial and phage proteins. Phage 7201 of *S. thermophilus* has a DnaD-like domain that is linked to a RepA-like domain thus far associated with the initiation of DNA replication, and as such, it is likely that such a domain is in fact an essential common structural module in the primosomal complex (Carneiro et al., 2006; Huang Y. H. et al., 2016).

### Phage Peptides Identified From the Analyzed *S. canis* Strain

Five peptides were determined from the analyzed *S. canis* strain ST10, including phage repressors of the Cro/CI type, a Gp58-like protein, a major tail protein, a phage integrase and an uncharacterized protein peptide. The peptides from *S. canis* strain ST10 were found to belong to three different described phages (Supplementary Table S1 in Supplementary Data 1). The function of these proteins was described above.

### Phage Peptides Identified From the Analyzed *S. gallolyticus* Strains

The *S. gallolyticus* strains analyzed were ST12 and ST13. Four peptides were identified from strain ST12, and six peptides were identified from strain ST13. Two ST12 peptides shared the same peptide sequence with two described phages in the NCBI database (Supplementary Table S1 in Supplementary Data 1), and the same applied for strain ST13.

Among the ST12 peptides, a phage endopeptidase peptide and an uncharacterized protein phage from phage phiARI0131-1 were determined. Additionally, a cytosine methyltransferase peptide was identified in strain ST12. Cytosine methyltransferase is a type II DNA methyltransferase (MTase) C5-methylcytosine (Class III) currently found in the prokaryotic world and associated with a restriction endonuclease that forms a restriction modification (R/M) system, thus protecting bacterial cells from foreign DNA. These methyltransferases have also been found in phage genomes, and they exhibit the ability to confer protection from REases (cognate restriction endonuclease) of their bacterial host(s) (Murphy et al., 2013). Furthermore, for *S. gallolyticus* ST12, a PBSX family phage terminase peptide was identified, and it is involved in DNA packaging and has also been implicated in endonuclease, ATPase, and double-stranded DNA binding activities (Gual et al., 2000). A PBSX family peptide, a phage endopeptidase peptide, a head tail connector peptide, a phage tape measure peptide and two uncharacterized protein peptides were identified in strain ST13.

Additionally, different strains from different species presented peptide sequences that belong to the same described phages (Supplementary Table S1 in Supplementary Data 1).

### *Streptococcus* spp. Phage Genome Comparison and Their Relatedness

**FIGURE 2:**
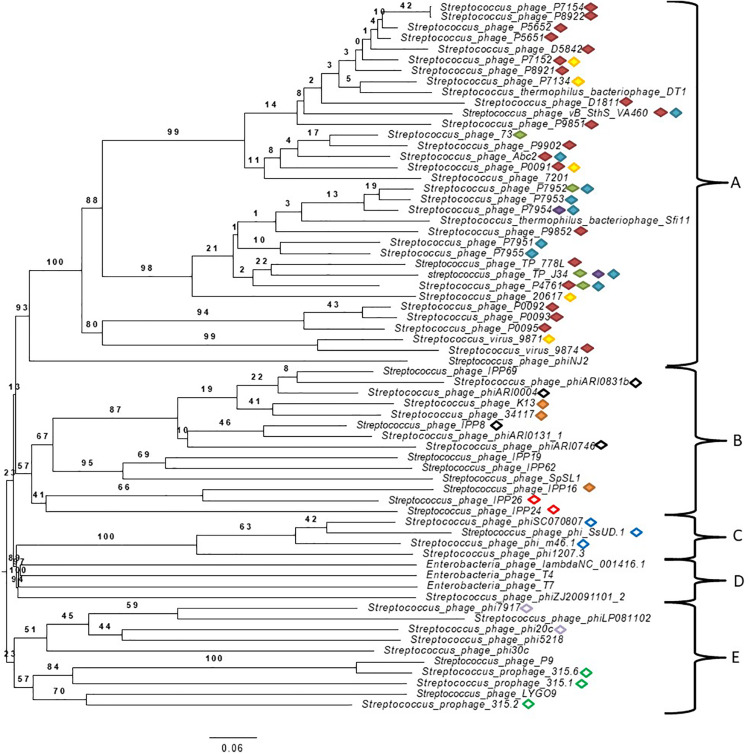
Phylogenetic tree generated by VICTOR using the complete genomic sequences of *Streptococcus* virus phiAbc2 (NC_013645.1), *Streptococcus* phage 315.1 (NC_004584.1), *Streptococcus* phage 34117 (FR671407.1), *Streptococcus* phage 73 (KT717083.1), *Streptococcus* phage D5842 (MH000602.1), *Streptococcus* phage IPP26 (KY065467.1), *Streptococcus* phage IPP8 (KY065450.1), *Streptococcus* phage K13 (NC_024357.1), *Streptococcus* phage P0091 (KY705251.1), *Streptococcus* phage P0092 (KY705252.1), *Streptococcus* phage P0093 (KY705253.1), *Streptococcus* phage P0095 (KY705255.1), *Streptococcus* phage P4761 (KY705258.1), *Streptococcus* phage P5651 (KY705260.1), *Streptococcus* phage P5652 (KY705261.1), *Streptococcus* phage P7134 (KY705264.1), *Streptococcus* phage P7152 (KY705266.1), *Streptococcus* phage P7154 (KY705267.1), *Streptococcus* phage P7951 (KY705277.1), *Streptococcus* phage P7952 (KY705278.1), *Streptococcus* phage P7953 (KY705279.1), *Streptococcus* phage P7954 (KY705280.1), *Streptococcus* phage P7955 (KY705281.1), *Streptococcus* phage P8921 (KY705282.1), *Streptococcus* phage P8922 (KY705283.1), *Streptococcus* phage P9851 (KY705284.1), *Streptococcus* phage P9852 (KY705285.1), *Streptococcus* phage P9902 (KY705289.1), *Streptococcus* phage phi7917 (KC348601.1), *Streptococcus* phage phiARI0004 (NC_031920.1), *Streptococcus* phage phiARI0746 (NC_031907.1), *Streptococcus* phage phi-m46.1 (FM864213.1), *Streptococcus* phage phiSC070807 (KT336321.1), *Streptococcus* phage phiZJ20091101-2 (KX077893.1), *Streptococcus* phage TP-778L (NC_022776.1), *Streptococcus* phage TP-J34 (HE861935.1), *Streptococcus* phage TP-J34 (HE861935.1), *Streptococcus* virus 7201 (NC_002185.1), *Streptococcus* virus 9871 (NC_031069.1), *Streptococcus* virus 9874 (NC_031023.1), *Streptococcus* virus DT1 (AF085222.2), *Streptococcus* phage 20617 (NC_023503.1), *Streptococcus* phage 315.2 (NC_004585.1), *Streptococcus* phage 315.6 (NC_004589.1), *Streptococcus* phage D1811 (MH000604.1), *Streptococcus* phage IPP16 (KY065457.1), *Streptococcus* phage IPP19 (Y065460.1), *Streptococcus* phage IPP24 (KY065465.1), *Streptococcus* phage IPP62 (KY065498.1), *Streptococcus* phage IPP69 (KY065505.1), *Streptococcus* phage LYGO9 (JX409894.1), *Streptococcus* phage P9 (NC_009819.1), *Streptococcus* phage phi1207.3 (AY657002.1), *Streptococcus* phage phi20c (KC348598.1), *Streptococcus* phage phi30c (KC348599.1), *Streptococcus* phage phi5218 (KC348600.1), *Streptococcus* phage phiARI0131-1 (NC_031901.1), *Streptococcus* phage phiARI0831b (KT337369.1), *Streptococcus* phage phiLP081102 (KX077890.1), *Streptococcus* phage phiNJ2 (NC_019418.1), *Streptococcus* phage phi-SsUD.1 (FN997652.1), *Streptococcus* phage SpSL1 (NC_027396.1), *Streptococcus* phage vB_SthS_VA460 (MG708275.1), *Streptococcus* virus Sfi11 (NC_002214.1) and phage Lambda (NC_001416.1), phage T4 (NC_000866.4) and phage T7 (NC_001604.1) as well-known bacteriophages of different studied families. Specific cluster peptides are represented by different color forms: 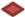; red filled diamond YPNSFTAYIMDVKGC*K (cluster A specific), 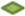; green filled diamond VKEKIAAIVNDEELLAK (cluster A specific), 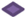; purple filled diamond ISGITLSDTNVVAGNLISSSEHFVQM*LSNIK (cluster A specific), 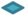; blue filled diamond FQLALLNTTTTFAQYQCSAYIDFEGQR (cluster A specific), 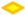; yellow filled diamond DNVLIETVKM*QGEQAM*QLVK (cluster A specific), 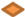; orange filled diamond TTTELTTITHTVGDIQKEMVRM*NDR (cluster B specific), 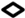; black outlined diamond M*LANGITLSYGESKETYTKLVGLK (cluster B specific), 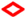; red outlined diamond LSDKPKSYTEILNNIINSQK (cluster B specific), 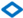; blue outlined diamond ITTGIISAARIGAEAITADKLK (cluster C specific), 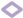; gray outlined diamond HAQGDVGWNINSVTQRIVPANINAESEIWSK (cluster E specific), 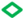; green outlined diamond TLITMTEQIKNLTDDVK (cluster E specific). (M*methionine oxidation; C* carbamidomethylation of Cys).

A phylogenomic tree of the complete genome of *Streptococcus* spp. phages from the NCBI database (accession numbers can be found in Supplementary Table S2 in Supplementary Data 1) with 100% similarity to those found in this study was built (Figure 2). Many phages found in this study were classified in the order *Caudovirales* but not in families, and some were unclassified. Genomes of well-known phages of different families including *Siphoviridae*, *Myoviridae*, and *Podoviridae*, such as phage lambda, T4 and T7, respectively, were added for comparison purposes. The analysis of genomes showed five well-defined clusters. To the best of our knowledge, this is the first time that phages from mastitis-causing streptococci were grouped in a phylogenetic tree. Two principal branches separated clusters A, B, C, and D from cluster E. Clusters A and B were located in the same secondary branch, and the same occurred with clusters C and D. Cluster A was formed by *Streptococcus/Siphoviridae* viruses, most of which were classified as Sfi21 dt1 viruses. Cluster B was formed by unclassified *Streptococcus* phages, and 2 *Streptococcus* phages were classified as *Siphoviridae*. Cluster C was formed by unclassified *Streptococcus* phages, and cluster D, which is related to cluster C, was formed by phage lambda, T4 and T7. Finally, cluster E was formed by *Streptococcus/Podoviridae* phages and unclassified *Streptococcus* phages.

Peptide biomarkers were found in related *Streptococcus* phages (Table 2) that were located closely in the phylogenomic tree (Figure 2). The peptides YPNSFTAYIM^∗^DVKGC^∗^K and VKEKIAAIVNDEELLAK were found in 23 and 4 *Streptococcus* phages of cluster A, respectively. The peptide ISGITLSDTNVVAGNLISSSEHFVQM^∗^LSNIK was also found in 2 closely related phages (*Streptococcus* phage P7954 and *Streptococcus* phage TP-J34) of cluster A. The peptides FQLALLNTTTTFAQYQC^∗^SAYIDFEGQR and DNVLIETVKM^∗^QGEQAM^∗^QLVK were found in 9 and 5 phages of cluster A, respectively. The peptide TTTELTTITHTVGDIQKEM^∗^VRM^∗^NDR was found in 3 *Streptococcus* phages (*Streptococcus* phage IPP16, *Streptococcus* phage K13, and *Streptococcus* phage 34117) of cluster B. The peptide M^∗^LANGITLSYGESKETYTKLVGLK was found in 4 phages of the firs branch of cluster B, and the peptide LSDKPKSYTEILNNIINSQK was found in 2 phages (*Streptococcus* phage IPP24 and *Streptococcus* phage IPP26) of the second branch of cluster B. The peptide ITTGIISAARIGAEAITADKLK was found in 3 (*Streptococcus* phage phi-SsUD.1, *Streptococcus* phage phi-m46.1, and *Streptococcus* phage phiSC070807) of 4 *Streptococcus* phages of cluster C. The peptide HAQGDVGWNINSVTQRIVPANINAESEIWSK was found in 2 phages (*Streptococcus* phage phi20c and *Streptococcus* phage phi7917) of the first branch of cluster E, and the peptide TLITM^∗^TEQIKNLTDDVK was found in 3 phages (*Streptococcus* phage 315.2 and *Streptococcus* phage 315.6, *Streptococcus* phage 315.1) of the second branch.

**TABLE 2:** Phage biomarker peptides that belong to bacteriophages, and phylogenomic tree clusters are located.

Protein	Peptide	Phages	Cluster located
Uncharacterized phage protein	YPNSFTAYIM*DVKGC*K	*Streptococcus* phage D1811 *Streptococcus* phage TP-778L *Streptococcus* phage D5842 *Streptococcus* phage vB_SthS_VA460 *Streptococcus* phage P9902 *Streptococcus* phage P9852 *Streptococcus* phage P8922 *Streptococcus* phage P9851 *Streptococcus* phage P8921 *Streptococcus* phage P7152 *Streptococcus* phage P7154 *Streptococcus* phage P7151 *Streptococcus* phage P5652 *Streptococcus* phage P5651 *Streptococcus* phage P4761 *Streptococcus* phage P0095 *Streptococcus* phage P0093 *Streptococcus* phage P0091 *Streptococcus* phage P0092 *Streptococcus* virus 9874 *Streptococcus* virus DT1 *Streptococcus* virus 7201 *Streptococcus* virus phiAbc2.	Cluster A
Uncharacterized phage protein	VKEKIAAIVNDEELLAK	*Streptococcus* phage P4761 *Streptococcus* phage 73 *Streptococcus* phage TP-J34 *Streptococcus* phage P7952	Cluster A
Minor phage structural protein	ISGITLSDTNVVAGNLISSSEHFVQM*LSNIK	*Streptococcus* phage P7954 *Streptococcus* phage TP-J34	Cluster A
Phage tail protein	FQLALLNTTTTFAQYQC*SAYIDFEGQR	*Streptococcus* phage vB_SthS_VA460 *Streptococcus* phage P7953 *Streptococcus* phage P7952 *Streptococcus* phage P7954 *Streptococcus* phage P7951 *Streptococcus* virus phiAbc2 *Streptococcus* phage P7955 *Streptococcus* phage TP-J34 *Streptococcus* phage P4761	Cluster A
Uncharacterized phage protein	DNVLIETVKM*QGEQAM*QLVK	*Streptococcus* phage 20617 *Streptococcus* phage P7152 *Streptococcus* phage P7134 *Streptococcus* virus 9871 *Streptococcus* phage P0091	Cluster A
Uncharacterized phage protein	TTTELTTITHTVGDIQKEM*VRMNDR	*Streptococcus* phage IPP16 *Streptococcus* phage K13 *Streptococcus* phage 34117	Cluster B
Major tail protein	M*LANGITLSYGESKETYTKLVGLK	*Streptococcus* phage phiARI0831b *Streptococcus* phage phiARI0004 *Streptococcus* phage phiARI0746 *Streptococcus* phage IPP8	ClusterB
Phage GDSL-like family lipase/acylhydrolase	LSDKPKSYTEILNNIINSQK	*Streptococcus* phage IPP24 *Streptococcus* phage IPP26	Cluster B
Endopeptidase/matrix-binding protein EbhB	ITTGIISAARIGAEAITADKLK	*Streptococcus* phage phi-SsUD.1 *Streptococcus* phage phi-m46.1 *Streptococcus* phage phiSC070807	Cluster D
Gp58-like protein	HAQGDVGWNINSVTQRIVPANINAESEIWSK	*Streptococcus* phage phi20c *Streptococcus* phage phi7917	Cluster E
Uncharacterized phage protein	TLITM*TEQIKNLTDDVK	*Streptococcus* phage 315.2 *Streptococcus* phage 315.6 *Streptococcus* phage 315.1	Cluster E

Relations between specific phage biomarker peptides and phylogenomic tree clusters. (M* methionine oxidation; C* carbamidomethylation of Cys).

In addition, a correlation that related all peptides found in bacteriophages with 100% similarity and with bacterial species for each cluster was inferred. The results showed that the clustered phages were related to specific species of *Streptococcus*. Cluster A phage peptides were found to be related mainly to *S. thermophilus.* All cluster B phage peptides were found to be related mainly to *S. pneumoniae.* Cluster C phage peptides were found to be mainly related to *S. uberis* and *Streptococcus suis*. Cluster E phage peptides were found in different *Streptococcus* species. However, the bacterial species *S. uberis* and *S. parauberis* were often found to be related in all clusters of the phylogenomic tree. These results support previously reported data on studied phages and where they were isolated from Brueggemann et al. (2017), McDonnell et al. (2017).

**FIGURE 3:**
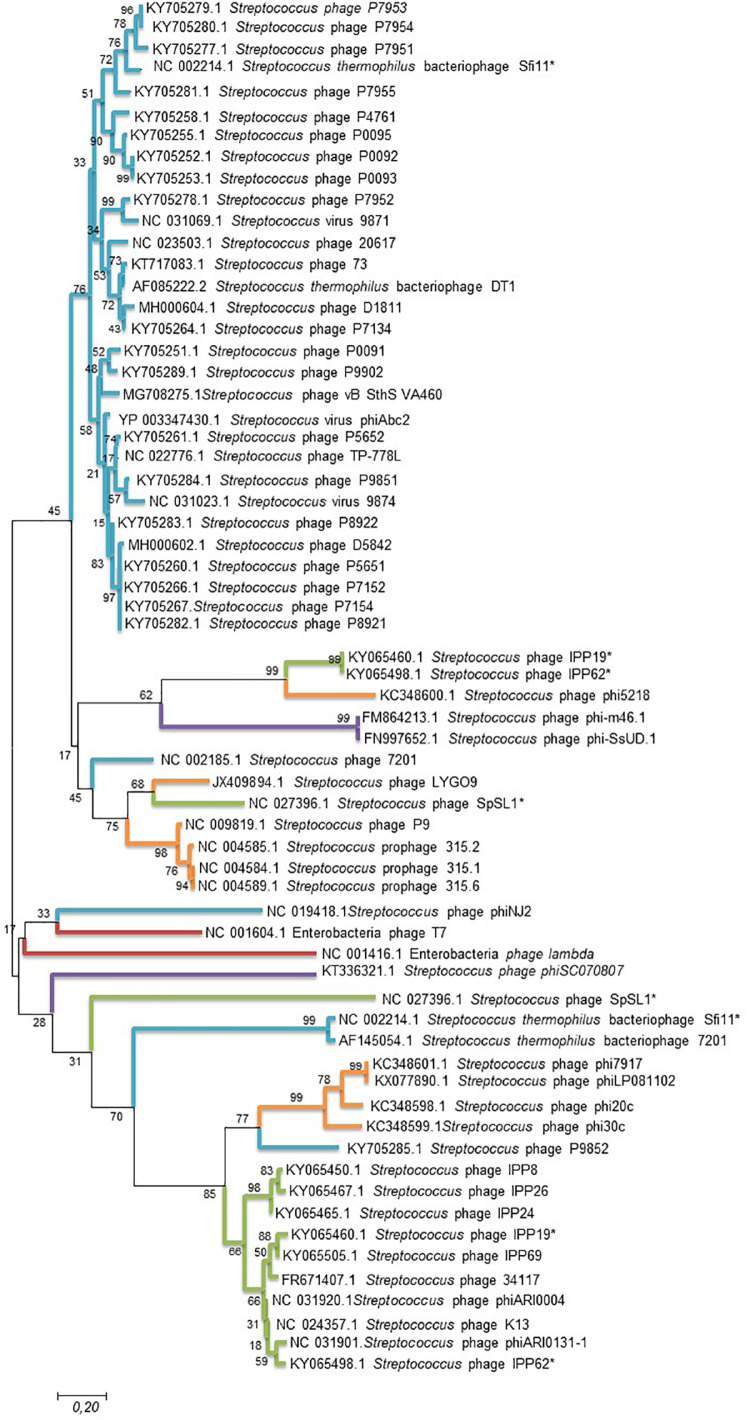
Maximum likelihood phylogenetic tree based on sequences of the *holin* gene of *Streptococcus* phages. *Streptococcus* phages of the different clusters displayed in the phylogenetic tree generated by VICTOR (Figure 2) are represented by different color branches; blue; cluster A, green; cluster B, purple; cluster C, red; cluster D and, orange; cluster E. The significance of each branch is indicated by a bootstrap value calculated as a percentage for 1,000 subsets. Bar, 20 nt substitutions per 100 nt. *two holin-like sequences.

To further analyze phage clustering, we selected the holin gene to construct a phylogenetic relationship tree and compared the phylogenomic relationship among phages with 100% similarity to the peptides. The holin protein is essential for host lysis and constitutes one of the most diverse functional proteins in phage genomes analyzed previously (Wang et al., 2000; Goerke et al., 2009). The comparison of the *holin* gene sequences of the studied bacteriophages (Figure 3) showed that the phylogenomic and phylogenetic clusters were similar (Figure 3). Cluster A of the phylogenomic tree was well defined in the holin tree. Previous analyses revealed two holin-like sequences, class I and class II, immediately upstream of the endolysin gene (Wang et al., 2000). This was observed for some of the bacteriophage holins that appeared in different clusters of the phylogenetic tree.

## Discussion

Proteomics techniques using matrix-assisted laser desorption/ionization time of flight mass spectrometry (MALDI-TOF MS) and liquid chromatography-electrospray ionization-tandem mass spectrometry (LC-ESI-MS/MS) instruments have been used for the analysis and detection of pathogenic bacterial pathogen strains based on specific diagnostic peptides (Lasch et al., 2009; Böhme et al., 2011, 2012; Branquinho et al., 2014; Quintela-Baluja et al., 2014; Calo-Mata et al., 2016; Pfrunder et al., 2016; Carrera et al., 2017). In addition, LC-ESI-MS/MS techniques have been employed for the identification and detection of bacterial bacteriophages such as the bacteriophage lambda (Serafim et al., 2017).

LC-MS/MS-based methods offer several advantages compared with other approaches because one can directly identify bacteriophage peptides without the necessity of inferring conclusions based on other approaches such as genomics tools. Additionally, in this study, we implemented a precise method that may be useful for further research and analysis, without the requirement of growing bacteria; instead, the samples can be collected directly from food. The total time estimated to complete the analysis since the beginning of the experiments is 3 days, and the direct analysis from foodstuffs should take less than 1 day.

Phage peptides such as M^∗^ATNLGQAYVQIM^∗^PSAK could be good biomarkers because they are unique and specific for a particular bacterial species, in this case for *S. agalactiae.* Additionally, some determined phage-origin peptides in this study could be useful as specific biomarker peptides for the distinct detection of pathogenic bacteria and/or their phages in a sample. As bacteriophages are signal amplifiers because of their life cycle, bacterial peptide and phage peptide detection is significant for the identification of pathogens.

As presented in this manuscript, LC-ESI-MS/MS analysis is a good strategy for the rapid characterization of phage-origin proteins present in *Streptococcus* spp. In addition, the peptides in this study may be very useful as peptide biomarkers for the identification and characterization of mastitis-causing *Streptococcus* spp.

Additionally, the results show that a specific peptide biomarker is present in near-related phages, and bacteriophages that present the same peptides as specific *Streptococcus* spp. are located very close in the phylogenomic tree. This suggests that a link exists between phage phylogeny and bacteriophages that could infect the same bacterial species. Due to bacteriophage activity against host species, there is a high possibility that phages will be effective against related host species within the genus *Streptococcus*. These data support the use of bacteriophages in what is known as “phage therapy” to control pathogenic bacterial populations.

Moreover, this study shows how phylogenomic trees based on genome analysis provide useful information and corroborates previous investigations. These previous studies suggested that this type of analysis as well as phylogenetic studies is an appropriate tool for studying and reconstructing the viral history of phages, and this tool may be considered a significant complement to virus classification based on properties such as morphology, genome composition (DNA/RNA), orientation (+/− sense), segmentation, gene sets and replication strategies (Simmonds, 2015). On the other hand, phage genomic content is not currently well known (Argov et al., 2017), making phage analysis more difficult. The first priority then must be guided by the acquisition of large amounts of data for phage-infecting bacteria (Brüssow and Desiere, 2001).

Finally, it is known that bacteria-phage interactions are mutually beneficial, and their coevolution maintains phenotypic and genetic diversity by contributing dramatically to lateral gene transfer (Koskella and Brockhurst, 2014). As the majority of bacteria contain bacteriophage DNA in their genomes, it is expected that additional and novel interactions will be observed in the future, thus contributing to a better understanding of bacteriophage biology (Argov et al., 2017).

## Data Availability Statement

The datasets generated for this study can be found in the Uniprot and GenBank: complete genomic sequences of *Streptococcus* virus phiAbc2 (NC_013645.1), *Streptococcus* phage 315.1 (NC_004584.1), *Streptococcus* phage 34117 (FR671407.1), *Streptococcus* phage 73 (KT717083.1), *Streptococcus* phage D5842 (MH000602.1), *Streptococcus* phage IPP26 (KY065467.1), *Streptococcus* phage IPP8 (KY065450.1), *Streptococcus* phage K13 (NC_024357.1), *Streptococcus* phage P0091 (KY705251.1), *Streptococcus* phage P0092 (KY705252.1), *Streptococcus* phage P0093 (KY705253.1), *Streptococcus* phage P0095 (KY705255.1), *Streptococcus* phage P4761 (KY705258.1), *Streptococcus* phage P5651 (KY705260.1), *Streptococcus* phage P5652 (KY705261.1), *Streptococcus* phage P7134 (KY705264.1), *Streptococcus* phage P7152 (KY705266.1), *Streptococcus* phage P7154 (KY705267.1), *Streptococcus* phage P7951 (KY705277.1), *Streptococcus* phage P7952 (KY705278.1), *Streptococcus* phage P7953 (KY705279.1), *Streptococcus* phage P7954 (KY705280.1), *Streptococcus* phage P7955 (KY705281.1), *Streptococcus* phage P8921 (KY705282.1), *Streptococcus* phage P8922 (KY705283.1), *Streptococcus* phage P9851 (KY705284.1), *Streptococcus* phage P9852 (KY705285.1), *Streptococcus* phage P9902 (KY705289.1), *Streptococcus* phage phi7917 (KC348601.1), *Streptococcus* phage phiARI0004 (NC_031920.1), *Streptococcus* phage phiARI0746 (NC_031907.1), *Streptococcus* phage phi-m46.1 (FM864213.1), *Streptococcus* phage phiSC070807 (KT336321.1), *Streptococcus* phage phiZJ20091101-2 (KX077893.1), *Streptococcus* phage TP-778L (NC_022776.1), *Streptococcus* phage TP-J34 (HE861935.1), *Streptococcus* phage TP-J34 (HE861935.1), *Streptococcus* virus 7201 (NC_002185.1), *Streptococcus* virus 9871 (NC_031069.1), *Streptococcus* virus 9874 (NC_031023.1), *Streptococcus* virus DT1 (AF085222.2), *Streptococcus* phage 20617 (NC_023503.1), *Streptococcus* phage 315.2 (NC_004585.1), *Streptococcus* phage 315.6 (NC_004589.1), *Streptococcus* phage D1811 (MH000604.1), *Streptococcus* phage IPP16 (KY065457.1), *Streptococcus* phage IPP19 (Y065460.1), *Streptococcus* phage IPP24 (KY065465.1), *Streptococcus* phage IPP62 (KY065498.1), *Streptococcus* phage IPP69 (KY065505.1), *Streptococcus* phage LYGO9 (JX409894.1), *Streptococcus* phage P9 (NC_009819.1), *Streptococcus* phage phi1207.3 (AY657002.1), *Streptococcus* phage phi20c (KC348598.1), *Streptococcus* phage phi30c (KC348599.1), *Streptococcus* phage phi5218 (KC348600.1), *Streptococcus* phage phiARI0131-1 (NC_031901.1), *Streptococcus* phage phiARI0831b (KT337369.1), *Streptococcus* phage phiLP081102 (KX077890.1), *Streptococcus* phage phiNJ2 (NC_019418.1), *Streptococcus* phage phi-SsUD.1 (FN997652.1), *Streptococcus* phage SpSL1 (NC_027396.1), *Streptococcus* phage vB_SthS_VA460 (MG708275.1), *Streptococcus* virus Sfi11 (NC_002214.1), and phage Lambda (NC_001416.1), phage T4 (NC_000866.4), phage T7 (NC_001604.1).

## Author Contributions

All authors listed have made a substantial, direct and intellectual contribution to the work, and approved it for publication.

## Conflict of Interest

The authors declare that the research was conducted in the absence of any commercial or financial relationships that could be construed as a potential conflict of interest.

## Footnotes

1 http://ggdc.dsmz.de/victor.php
