# Supplementary material for: Characterization of Bacteriophage Peptides of Pathogenic Streptococcus by LC-ESI-MS/MS: Bacteriophage Phylogenomics and Their Relationship to Their Host
Source: Front Microbiol. 2020 Jun 9;11:1241. doi: 10.3389/fmicb.2020.01241 (PMC7296060; doi:10.3389/fmicb.2020.01241)
Supplement: Supplementary file 1 [file Data_Sheet_1.PDF]

Supplementary Material

Table 1 Supplemental Data 1. Phage-origin peptides identified in *Streptococcus* spp. strains.

Strain	Protein	Peptide	100% bacteria homology with protein NCBI database	100% phage homology with protein NCBI database
ST1_ATCC 19436	Gp58-like protein	M*SSAESAITQNATEISK	*Streptococcus suis*	*Streptococcus* phage phi30c
ST1_ATCC 19436	RecT protein	NSSSGNLLSQC*TDESVYISLLDM*VAQGLSPAK K	*Streptococcus agalactiae*	*Streptococcus* phage LYGO9
ST1_ATCC 19436	Tape measure protein	LNSTFRLQESQLGSNASESEK	*Streptococcus pyogenes*<br>*Streptococcus gordonii*	*Streptococcus* phage phi1207.3
ST1_ATCC 19436	Phage head protein	WSGDNYSGRIWDNTGELAR	*Streptococcus anginosus*<br>*Streptococcus intermedius*	
ST1_ATCC 19436	Phage head protein	LSM*AENGFTM*FDIIPEPKAC*DICK	*Streptococcus pyogenes*<br>*Streptococcus dysgalactiae*	
ST1_ATCC 19436	Phage tail protein	SNDLGLLLERERSIK	*Streptococcus pyogenes*<br>*Streptococcus equi subsp. equi*<br>*Streptococcus dysgalactiae*	

ST1_ATCC 19436	Uncharacterized phage protein	RELVDKEDTQGLAIDAQFLYDC*K	*Streptococcus suis*	
ST1_ATCC 19436	Phage tail protein	RSVM*ELSYTLYLVKPSEEQLLSFLKLFLK	*Streptococcus* suis	*Streptococcus* phage phi-m46.1
ST1_ATCC 19436	Endopeptidase/ matrix- binding protein EbhB	ITTGIISAARIGAEAITADKLK	*Streptococcus suis*	*Streptococcus* phage phi-SsUD.1<br>*Streptococcus* phage phi-m46.1<br>*Streptococcus* phage phiSC070807
ST1_ATCC 19436	Phage minor structural protein GP20 family	LIDAELTKAGVRDAEIFGK	*Streptococcus mitis*	
ST1_ATCC 19436	Uncharacterized phage protein	VKEKIAAIVNDEELLAK		*Streptococcus* phage P4761, *Streptococcus* phage 73, *Streptococcus* phage TP-J34, *Streptococcus* phage P7952
ST1_ATCC 19436	Uncharacterized phage protein	M*EKLIESLDNLIM*IVK	*Streptococcus pneumoniae*	*Streptococcus* phage IPP19
ST2_CECT 183T	Uncharacterized phage protein	TLITM*TEQIKNLTDDVK	*Streptococcus pyogenes*	*Streptococcus* phage 315.2, *Streptococcus* phage 315.6, *Streptococcus* phage 315.1
ST2_CECT 183T	Phage tail protein	LAFSGAGNAVDVLKNVFSLAWM*GIQDVVKVA K	*Streptococcus suis*	
ST2_CECT 183T	Phage envelope protein	AM*DGM*YEHINAFNYKAEDVIGVGKTK	*Streptococcus suis*	*Streptococcus* phage phiNJ2
ST2_CECT 183T	Minor phage structural protein	ISGITLSDTNVVAGNLISSSEHFVQM*LSNIK		*Streptococcus* phage P7954, *Streptococcus* phage TP-J34
ST2_CECT 183T	Phage replication protein	TFKGINTNNINTNISINNK	*Streptococcus canis*	

ST2_CECT 183T	Phage-related chromosomal island protein	TWLDDAVSVDEAM*ALPENK	*Streptococcus pneumoniae*	
ST2_CECT 183T	Tail fiber pblA protein	M*ATNLGQAYVQIM*PSAK	*Streptococcus agalactiae*	
ST3_CECT 926	Head-tail connector protein	TEGGIEVVYGENGLSTGLLQRIRM*HR	*Streptococcus pneumoniae*	*Streptococcus* phage IPP69
ST3_CECT 926	Major tail protein	M*LANGITLSYGESKETYTKLVGLK	*Streptococcus pneumoniae*	*Streptococcus* phage phiARI0831b, *Streptococcus* phage phiARI0004, *Streptococcus* phage phiARI0746, *Streptococcus* phage IPP8
ST3_CECT 926	Phage tail assembly chaperone protein	LDDEAYDKLLDLDNERTQEIAEK	*Streptococcus salivarius*	*Streptococcus* virus 7201
ST3_CECT 926	Phage integrase	TKPVTVYNDNK	*Streptococcus Parauberis*<br>*Streptococcus agalactiae*	
ST3_CECT 926	Phage capsid protein	DDIKGSVELM*LSVWGVQKK	*Streptococcus pyogenes*	
ST3_CECT 926	Phage repressor, Cro/CI family	QASNLSQKDFFEKIVK	*Streptococcus suis*	
ST3_CECT 926	Phage-like HicB family antitoxin	NEDFEM*VYDSSKSFISM*VM*VDVAKYLGSQEPVK	*Streptococcus suis*	
ST4_DSM 6631	Phage GDSL-like family lipase/acylhydrolase	LSDKPKSYTEILNNIINSQK	*Streptococcus pneumoniae*	*Streptococcus* phage IPP24, *Streptococcus* phage IPP26

ST4_DSM 6631	Phage protein (DUF1372 domain)	DVVDGHYYVEVKPYGKFLVTK	*Streptococcus suis*	
ST4_DSM 6631	Phage tail protein	FQLALLNTTTTFAQYQC*SAYIDFEGQR	*Streptococcus thermophilus*	*Streptococcus* phage vB_SthS_VA460, *Streptococcus* phage P7953, *Streptococcus* phage P7952, *Streptococcus* phage P7954, *Streptococcus* phage P7951, *Streptococcus* virus phiAbc2, *Streptococcus* phage P7955, *Streptococcus* phage TP-J34, *Streptococcus* phage P4761
ST4_DSM 6631	Uncharacterized phage protein	DNVLIETVKM*QGEQAM*QLVK		*Streptococcus* phage 20617, *Streptococcus* phage P7152, *Streptococcus* phage P7134, *Streptococcus* virus 9871, *Streptococcus* phage P0091,
ST5_DSM 6632	Phage repressor, Cro/CI family	KERNLTQEEM*ADK	*Streptococcus suis*,	*Streptococcus* phage phiLP081102
ST5_DSM 6632 and ST7_USC 3	Phage tail fiber PblB	DNFAISIKRNR	*Streptococcus mitis*<br>*Streptococcus pseudopneumoniae*<br>*Streptococcus pneumoniae*	*Streptococcus* phage SpSL1
ST5_DSM 6632	Uncharacterized phage protein	TTTELTTITHTVGDIQKEM*VRM*NDR	*Streptococcus pneumoniae*<br>*Mycobacterium tuberculosis*	*Streptococcus* phage IPP16, *Streptococcus* phage K13, *Streptococcus* phage 34117
ST6_USC 1	Uncharacterized phage protein	TEQLPFANTNLK	*Streptococcus pyogenes*	
ST7_USC 3	Major capsid protein	KM*VEVGELEENPELGK	*Streptococcus suis*<br>*Streptococcus plurextorum*	

ST7_USC 3	DnaD and phage-associated domain-containing protein	IEEVKEEKISTSSQNSQNIK	*Streptococcus henryi*	
ST7_USC 3	Tape measure protein	VEIVQEKSNEVM*EKAIDAM*GR	*Streptococcus lutetiensis*<br><br>*Streptococcus equinus*	
ST7_USC 3	Phage integrase	TLAKGEDNKLIFQTPK	*Streptococcus mitis*<br><br>*Streptococcus pseudopneumoniae*<br><br>*Streptococcus infantis*<br><br>*Streptococcus oralis*<br><br>*Streptococcus parasanguinis*	
ST8_USC 5	Phage protein, HK97 Gp10 family protein	M*AIKWQGM*EKLVATISNAHPK	*Streptococcus suis*	
ST8_USC 5	Gp348 protein, Mayor capsid protein	QLGTKLSYITGASGQSVALK		*Streptococcus* virus Sfi11
ST8_USC 5	Phage integrase	QLFSELGSNSPRIVFSTPTK	*Streptococcus parauberis*	
ST8_USC 5	Uncharacterized phage protein	NKGENQKMALSTR	*Streptococcus suis,*	*Streptococcus* phage phiZJ20091101-2

ST8_USC 5	Uncharacterized phage protein	YPNSFTAYIM*DVKGC*K	*Streptococcus thermophilus*	*Streptococcus* phage D1811, *Streptococcus* phage TP-778L, *Streptococcus* phage D5842, *Streptococcus* phage vB_SthS_VA460, *Streptococcus* phage P9902, *Streptococcus* phage P9852, *Streptococcus* phage P8922, *Streptococcus* phage P9851, *Streptococcus* phage P8921, *Streptococcus* phage P7152, *Streptococcus* phage P7154, *Streptococcus* phage P7151, *Streptococcus* phage P5652, *Streptococcus* phage P5651, *Streptococcus* phage P4761, *Streptococcus* phage P0095, *Streptococcus* phage P0093, *Streptococcus* phage P0091, *Streptococcus* phage P0092, *Streptococcus* virus 9874, *Streptococcus* virus DT1, *Streptococcus* virus 7201, *Streptococcus* virus phiAbc2.
ST9_USC 13	Prophage pi2 protein	M*DDLFDSHSIFFDKVETYISTEK	*Streptococcus oralis*<br>*Streptococcus mitis*	
ST9_USC 13	Uncharacterized phage protein	EVAKIRFEDNEYDVHTVK		*Streptococcus* phage phi5218
ST10_USC 52	Phage repressor, Cro/CI family	YPDRFIPFEDEPRIVGLVVGHFM*PVIGG	*Streptococcus salivarius*<br>*Streptococcus sanguinis*<br>*Streptococcus gordonii*	
ST10_USC 52 and ST14_USC 158	Gp58-like protein	HAQGDVGWNINSVTQRIVPANINAESEIWSK	*Streptococcus suis*	*Streptococcus* phage phi20c, *Streptococcus* phage phi7917

ST10_USC 52	Uncharacterized phage protein	AGNNEIVFIC*KC*IC*GNIIESYTSLLR	*Streptococcus parauberis*	
ST10_USC 52	Major tail protein	M*TVKGTALIGLKSVTIR	*Streptococcus pneumoniae*	*Streptococcus* phage IPP62
ST10_USC 52	Phage integrase family	RFLQQAIEKVIDSNGYITAK	*Streptococcus mitis*	
ST12_USC 83	Phage cytosine-specific methyltransferase	FLDLFAGIGGFRFGM*ESAGHEC*IGFC*EIDK	*Streptococcus pneumoniae*<br><br>*Streptococcus oralis,*	*Streptococcus* phage phiARI0746
ST12_USC 83	Phage endopeptidase	QKEFEETLRNLAIPEEAIK	*Streptococcus canis*	
ST12_USC 83	PBSX family phage terminase	LAQAQAEAGDQTAEAQVKAISEIIGKM*ENAVS	*Streptococcus oralis*	
ST12_USC 83	Uncharacterized protein phage phiARI0131-1	KDSVNGPAPIPAGTYPVVLKK	*Streptococcus pneumoniae*	*Streptococcus* phage phiARI0131-1
ST13_USC 84	PBSX family phage terminase	VGRSLKDSIFEDVK	*Streptococcus agalactiae*<br><br>*Enterococcus faecalis*	
ST13_USC 84	Phage endopeptidase	LVLTPTKAKM*EAQTFNLSK	*Streptococcus equi subsp. equi*<br><br>*Streptococcus equi subsp. zooepidemicus*<br><br>*Streptococcus equi*	*Streptococcus* phage P9
ST13_USC 84	Head tail connector protein	RSQEM*SKAPIYAIYK	*Streptococcus pneumoniae*<br><br>*Staphylococcus warneri*<br><br>*Staphylococcus* spp.	Uncultured Caudovirales phage

ST13_USC 84	Phage tail tape-measure protein	DDSDGAGSGGSGGGKGKGK	*Streptococcus oralis*	
ST13_USC 89	Uncharacterized phage protein	QM*KDYIVENQGLIGKR	*Streptococcus pneumoniae*	
ST13_USC 84	Uncharacterized phage protein	IENM*LLAQENRRLEEQAK	*Streptococcus suis,*	*Streptococcus* phage phi20c
ST14_CECT 758	Phage integrase family	QFVFTYNDRSNNINLPLHVDYLNYRM*K	*Streptococcus thermophilus*<br>*Streptococcus salivarius*	
ST14_ CECT 758	Phage tail tape-measure protein	KWAM*EYGLSTKSVNSGM*QELVK	*Streptococcus parauberis*	
ST14_ CECT 758	Phage repressor, Cro/CI family	KLAYVKPRM*GM*NAFGKPGLTYEGIGESK	*Streptococcus salivarius*<br>*Streptococcus anginosus*<br>*Streptococcus sanguinis*	
ST14_ CECT 758	Uncharacterized phage protein	KLETAVVM*LVAENAM*QAKALR	*Streptococcus pyogenes*	
ST14_ CECT 758	Gp58-like protein	HAQGDVGWNINSVTQRIVPANINAESEIWSK	*Streptococcus suis,*	*Streptococcus* phage phi20c,<br>*Streptococcus* phage phi7917

(M* methionine oxidation; C* carbamidomethylation of Cys)

Table 2 in Supplemental Data 1 Linage, authors and accession number of studied bacteriophages.

Phage name	Linage	Accession number	Authors
Streptococcus virus phiAbc2	dsDNA viruses, no RNA stage; *Caudovirales*; *Siphoviridae*; Sfi21dt1 virus	NC_013645.1	Guglielmotti et al., 2005
Streptococcus phage 315.1	dsDNA viruses, no RNA stage; *Caudovirales*; *Podoviridae*; unclassified *Podoviridae*	NC_004584.1	Beres et al., 2002
Streptococcus phage 34117	unclassified bacterial viruses	FR671407.1	Croucher et al., 2011
Streptococcus phage 73	dsDNA viruses, no RNA stage; *Caudovirales*; *Siphoviridae*; unclassified *Siphoviridae*	KT717083.1	Achigar et al., 2017
Streptococcus phage D5842	dsDNA viruses, no RNA stage; *Caudovirales*; *Siphoviridae*; Sfi21dt1 virus; unclassified Sfi21dt1 virus	MH000602.1	Hynes et al., 2018
Streptococcus phage IPP26	unclassified bacterial viruses	KY065467.1	Brueggemann et al., 2017
Streptococcus phage IPP8	unclassified bacterial viruses	KY065450.1	Brueggemann et al., 2017
Streptococcus phage K13	unclassified bacterial viruses	NC_024357.1	Croucher et al., 2014
Streptococcus phage P0091	dsDNA viruses no RNA stage; *Caudovirales*; *Siphoviridae*; Sfi21dt1 virus; unclassified Sfi21dt1 virus	KY705251.1	McDonnell et al., 2017
Streptococcus phage P0092	dsDNA viruses no RNA stage; *Caudovirales*; *Siphoviridae*	KY705252.1	McDonnell et al., 2017
Streptococcus phage P0093	dsDNA viruses no RNA stage; *Caudovirales*; *Siphoviridae*	KY705253.1	McDonnell et al., 2017

Streptococcus phage P0095	dsDNA viruses no RNA stage; *Caudovirales*; *Siphoviridae*	KY705255.1	McDonnell et al., 2017
Streptococcus phage P4761	dsDNA viruses no RNA stage; *Caudovirales*; *Siphoviridae*; Sfi21dt1 virus; unclassified Sfi21dt1 virus	KY705258.1	McDonnell et al., 2017
Streptococcus phage P5651	dsDNA viruses no RNA stage; *Caudovirales*; *Siphoviridae*; Sfi21dt1 virus; unclassified Sfi21dt1 virus	KY705260.1	McDonnell et al., 2017
Streptococcus phage P5652	dsDNA viruses no RNA stage; *Caudovirales*; *Siphoviridae*; Sfi21dt1virus; unclassified Sfi21dt1 virus	KY705261.1	McDonnell et al., 2017
Streptococcus phage P7134	dsDNA viruses no RNA stage; *Caudovirales*; *Siphoviridae*; Sfi21dt1virus; unclassified Sfi21dt1 virus	KY705264.1	McDonnell et al., 2017
Streptococcus phage P7151	dsDNA viruses no RNA stage; *Caudovirales*; *Siphoviridae*; Sfi21dt1 virus; unclassified Sfi21dt1 virus	KY705265.1	McDonnell et al., 2017
Streptococcus phage P7152	dsDNA viruses no RNA stage; *Caudovirales*; *Siphoviridae*; Sfi21dt1 virus; unclassified Sfi21dt1 virus	KY705266.1	McDonnell et al., 2017
Streptococcus phage P7154	dsDNA viruses no RNA stage; *Caudovirales*; *Siphoviridae*; Sfi21dt1 virus; unclassified Sfi21dt1 virus	KY705267.1	McDonnell et al., 2017
Streptococcus phage P7951	dsDNA viruses no RNA stage; *Caudovirales*; *Siphoviridae*; Sfi21dt1 virus; unclassified Sfi21dt1 virus	KY705277.1	McDonnell et al., 2017
Streptococcus phage P7952	dsDNA viruses no RNA stage; *Caudovirales*; *Siphoviridae*; Sfi21dt1 virus; unclassified Sfi21dt1 virus	KY705278.1	McDonnell et al., 2017
Streptococcus phage P7953	dsDNA viruses no RNA stage; *Caudovirales*; *Siphoviridae*; Sfi21dt1 virus; unclassified Sfi21dt1 virus	KY705279.1	McDonnell et al., 2017

Streptococcus phage P7954	dsDNA viruses no RNA stage; *Caudovirales*; *Siphoviridae*; Sfi21dt1 virus; unclassified Sfi21dt1 virus	KY705280.1	McDonnell et al., 2017
Streptococcus phage P7955	dsDNA viruses no RNA stage; *Caudovirales*; *Siphoviridae*; Sfi21dt1 virus; unclassified Sfi21dt1 virus	KY705281.1	McDonnell et al., 2017
Streptococcus phage P8921	dsDNA viruses no RNA stage; *Caudovirales*; *Siphoviridae*; Sfi21dt1 virus; unclassified Sfi21dt1 virus	KY705282.1	McDonnell et al., 2017
Streptococcus phage P8922	dsDNA viruses no RNA stage; *Caudovirales*; *Siphoviridae*; Sfi21dt1 virus; unclassified Sfi21dt1 virus	KY705283.1	McDonnell et al., 2017
Streptococcus phage P9851	dsDNA viruses no RNA stage; *Caudovirales*; *Siphoviridae*; Sfi21dt1 virus; unclassified Sfi21dt1 virus	KY705284.1	McDonnell al. 2017
Streptococcus phage P9852	dsDNA viruses no RNA stage; *Caudovirales*; *Siphoviridae*; Sfi21dt1 virus; unclassified Sfi21dt1 virus	KY705285.1	McDonnell et al., 2017
Streptococcus phage P9902	dsDNA viruses no RNA stage; *Caudovirales*; *Siphoviridae*; Sfi21dt1 virus; unclassified Sfi21dt1 virus	KY705289.1	McDonnell et al., 2017
Streptococcus phage phi7917	dsDNA viruses no RNA stage; *Caudovirales*; *Podoviridae*; unclassified *Podoviridae*	KC348601.1	Tang et al., 2013
Streptococcus phage phiARI0004	unclassified bacterial viruses	NC_031920.1	Croucher et al., 2016
Streptococcus phage phiARI0746	dsDNA viruses no RNA stage; *Caudovirales*; *Siphoviridae*; unclassified *Siphoviridae*	NC_031907.1	Croucher et al., 2016
Streptococcus phage phi-m46.1	unclassified bacterial viruses	FM864213.1	Brenciani et al., 2010

Streptococcus phage phiSC070807	dsDNA viruses no RNA stage; *Caudovirales*; unclassified *Caudovirales*	KT336321.1	Huang and Wang (unpublished)
Streptococcus phage TP-778L	dsDNA viruses no RNA stage; *Caudovirales*; *Siphoviridae*; unclassified *Siphoviridae*	NC_022776.1	Ali et al., 2014
Streptococcus phage TP-J34	dsDNA viruses no RNA stage; *Caudovirales*; *Siphoviridae*; unclassified *Siphoviridae*	HE861935.1	Neve et al., 1998
Streptococcus virus 7201	dsDNA viruses no RNA stage; *Caudovirales*; *Siphoviridae*; Sfi21dt1virus	NC_002185.1	Stanley et al., 2000
Streptococcus virus 9871	dsDNA viruses no RNA stage; *Caudovirales*; *Siphoviridae*; unclassified *Siphoviridae*	NC_031069.1	McDonnell et al., 2016
Streptococcus virus 9874	dsDNA viruses no RNA stage; *Caudovirales*; *Siphoviridae*; unclassified *Siphoviridae*	NC_031023.1	McDonnell et al., 2016
Streptococcus virus DT1	dsDNA viruses no RNA stage; *Caudovirales*; *Siphoviridae*; Sfi21dt1 virus	AF085222.2	Tremblay and Moineau 1999
Streptococcus phage 20617	unclassified bacterial viruses	NC_023503.1	Arioli et al., 2018
Streptococcus phage 315.2	dsDNA viruses no RNA stage; *Caudovirales*; *Podoviridae*; unclassified *Podoviridae*	NC_004585.1	Beres et al., 2002
Streptococcus phage 315.6	dsDNA viruses no RNA stage; *Caudovirales*; *Podoviridae*; unclassified *Podoviridae*	NC_004589.1	Beres et al., 2002
Streptococcus phage D1811	dsDNA viruses no RNA stage; *Caudovirales*; *Siphoviridae*; Sfi21dt1 virus; unclassified Sfi21dt1 virus	MH000604.1	Hynes et al., 2018
Streptococcus phage IPP16	unclassified bacterial viruses	KY065457.1	Brueggemann et al., 2017

Streptococcus phage IPP19	dsDNA viruses no RNA stage; *Caudovirales*; unclassified *Caudovirales*	Y065460.1	Brueggemann A B et al., 2017
Streptococcus phage IPP24	unclassified bacterial viruses	KY065465.1	Brueggemann et al., 2017
Streptococcus phage IPP62	unclassified bacterial viruses	KY065498.1	Brueggemann et al., 2017
Streptococcus phage IPP69	unclassified bacterial viruses	KY065505.1	Brueggemann et al., 2017
Streptococcus phage LYGO9	dsDNA viruses no RNA stage; *Caudovirales*; *Siphoviridae*; unclassified *Siphoviridae*	JX409894.1	Bai et al., 2016
Streptococcus phage P9	dsDNA viruses no RNA stage; *Caudovirales*; *Siphoviridae*; unclassified *Siphoviridae*	NC_009819.1	Tiwari et al., 2006
Streptococcus phage phi1207.3	dsDNA viruses no RNA stage; *Caudovirales*; unclassified *Caudovirales*	AY657002.1	Iannelli et al., 2014
Streptococcus phage phi20c	unclassified bacterial viruses	KC348598.1	Tang et al., 2013
Streptococcus phage phi30c	dsDNA viruses no RNA stage; *Caudovirales*; *Podoviridae*; unclassified *Podoviridae*	KC348599.1	Tang et al., 2013
Streptococcus phage phi5218	dsDNA viruses no RNA stage; *Caudovirales*; *Podoviridae*; unclassified *Podoviridae*	KC348600.1	Tang et al., 2013
Streptococcus phage phiARI0131-1	unclassified bacterial viruses	NC_031901.1	Croucher et al., 2016
Streptococcus phage phiARI0831b	unclassified bacterial viruses	KT337369.1	Croucher et al., 2016

Streptococcus phage phiLP081102	dsDNA viruses no RNA stage; *Caudovirales*; *Podoviridae*; unclassified *Podoviridae*	KX077890.1	Huang et al., 2016a
Streptococcus phage phiNJ2	dsDNA viruses no RNA stage; *Caudovirales*; *Siphoviridae*; unclassified *Siphoviridae*	NC_019418.1	Tang et al., 2013
Streptococcus phage phi-SsUD.1	dsDNA viruses no RNA stage; *Caudovirales*; unclassified *Caudovirales*	FN997652.1	Palmieri et al., 2011
Streptococcus phage SpSL1	dsDNA viruses no RNA stage; *Caudovirales*; *Siphoviridae*; unclassified *Siphoviridae*	NC_027396.1	Furi Clockie and Oggioni (Unpublished)
Streptococcus phage vB_SthS_VA460	dsDNA viruses no RNA stage; *Caudovirales*; *Siphoviridae*; Sfi21dt1 virus; unclassified Sfi21dt1 virus	MG708275.1	da Silva Duarte et al., 2018
Streptococcus virus Sfi11	dsDNA viruses no RNA stage; *Caudovirales*; *Siphoviridae*; Sfi11 virus	NC_002214.1	Lucchini et al., 1999b
Streptococcus phage phiZJ20091101-2	Viruses; unclassified bacterial viruses	KX077893.1	Huang et al., 2016a
